# The Book World of Medicine and Science

**Published:** 1906-03-31

**Authors:** 


### The Book World of Medicine and Science.

Chemistry of the Albumens : Ten lectures delivered in the Michaelmas Term in the Physiological Department of University College, London, by S. B. Schryver, D.Sc. Lond., Ph.D. Leipzig. Lecturer on Physiological Chemistry in University College, London. (London : John Murray. Pp. 191. Price 7s. 6d. net.)

We are pleased to have in the English language a work devoted entirely to the chemistry of the albumens, and while thinking that the book under review is less comprehensive in scope than Cohnheim's well-known "Chemie der Eiweissinkörper," which was published in 1900, we do not admit that this is necessarily a disadvantage. The volume comprises ten chapters, each corresponding to a lecture as delivered. The first chapter deals with the general properties and reactions of the albumens, which include the methods of precipitation and separation, and four very interesting pages upon the question of crystallisation. Lecture II. and Lecture III. are devoted to what are termed the degradation products of albumens. The nomenclature adopted by the author is the German, as he differentiates between a proteid and an albumen, the former applying only to those albumens which contain the "prosthetic groups" of Hoppe Seyler. Considerable attention is given to carbohydrate substances which have been obtained by hydrolysis of the albumens, a very complete account of this work, from Schutzenberger downwards, being given. Lectures IV. and V. are devoted to nucleic acid and its degradation products, amongst which may be mentioned uric acid and the purin bases. Lecture VI. contains an interesting account of the glyco-proteids or bodies from which a carbohydrate group can be easily split off upon hydrolysis. This group includes the mucins, mucoids and cartilage. To the general reader Lecture VII. will perhaps be the most attractive, as in it is considered the chemistry of Hæmoglobin and its chromatogenic group. Lecture VIII. deals with albumoses and peptones. Lectures IX. and X. treat of biochemical action in general, the most comprehensive meaning possible being attached to this term. Under this heading are included theories relating to immunity and toxicity. The book is unquestionably of value and interest. The work is thoroughly done, and whenever practical the formulæ for the bodies and reactions under consideration are given. Those who can appreciate it will certainly find much profit in this excellent work.

Potter's Cyclopædia of Botanical Drugs and Preparations. By R. C. Wren. (London : Potter and Clarke, Artillery Lane, E. Pp. 40+208.)

This book is intended as a "guide to all who use Botanical Drugs." It professes to give information in reference to "every herb in general use," and, as among its contents we find particulars of abscess root, worm bark, bouncing-bet, burning-bush, cocklebur, jack-in-the-pulpit, Jacob's ladder, life everlasting, and the toothache tree, its claim to completeness appears to have a substantial defence. Among what people these and their like are in "general use" we do not know, but from a list of books printed at the end of the volume we gather that they are part of the armamentarium of "the eclectic practitioner" and of those who adhere to "The Working Man's Model Family Botanic Guide; or Every Man his own Doctor." The medicinal virtues attributed to the several herbs are varied and numerous, and are presented in crisp, confident sentences. Thus Red Root is "astringent, expectorant, antispasmodic. Used internally for gonorrhœa, asthma (*sic*), bronchitis, and pulmonary complaints. For sores in the mouth it makes an excellent wash"; while Yellow Dock "can be freely used in rheumatism, skin diseases, bilious complaints, piles, bleeding of the lungs," etc. Lastly we get a number of "receipts of medicinal compounds," as an example of which we may quote an approved "Cough Powder" containing, in addition to other ingredients, polypody root, skunk cabbage, pleurisy root, and black cohosh! Evidently all this relates to a world largely unknown to the medical practitioner, and into which, in view of the brevity of human life, we cannot advise him further to penetrate.

A Practical Guide to the Administration of the "Nauheim" Treatment of Chronic Diseases of the Heart in England. By Leslie Thorne Thorne, M.D. Second Edition (London : Baillière Tindall, and Cox. 1906. Pp. 75, with 56 figures. Price 3s. 6d. net.)

Experience has approved the Nauheim method of treating chronic cardiac disorders by medicated baths, and the systematised exercises introduced by the late Dr. Augustus Schott and his brother, Professor Theodore Schott, are now recognised as important elements of the treatment. The season at Nauheim extends from the beginning of May till the end of September. Such points as are beneficial for the patients should be capable of being carried out at all times and in every place. That this may be the case, Dr. Thorne in his monograph clearly indicates. He writes as an enthusiastic believer in the efficacy of this treatment, but indicates the necessity for discrimination in the selection of cases. The action and administration of the baths are carefully described, but the most serviceable portion of the book consists of the description and illustrations of the exercises. The clinical records of a number of cases afford evidence that, given suitable conditions, the treatment may be of considerable benefit. This little work is likely to prove of much use to the general practitioner.

Clinical Obstetrics. By Robert Jardine, M.D., Edin., F.R.S.Edin. (London : Rebman. Price 15s. net.)

Dr. Jardine has dedicated this work to "Those obstetricians who are endeavouring by aseptic methods to do for midwifery what has already been done for surgery." In his introductory chapter he makes an eloquent appeal for more asepsis in midwifery; he points out that whereas surgery has been largely in the hands of specialists, men who have time for the study of details, midwifery as a whole has been done by general practitioners who in the rush of their work often forget that surgery has attained a degree of brilliance of which midwifery has fallen short. Throughout the book he shows how well asepsis may be attained. The author also recognises that the clinical teaching of this branch of medicine is deficient and has made his book essentially clinical; it is copiously illustrated both by cases and pictures, many of the latter being reproductions of photographs. In a work so full of useful information it would be difficult to pick out one part as more important than another, but perhaps the chapter devoted to eclampsia is one of the more interesting. The author recommends the use of large saline infusions, to each pint of which he adds a drachm of sodium acetate for its diuretic effect, and by this means he claims the mortality from eclampsia at the Glasgow Maternity Hospital has been reduced 50 per cent. The book is well written and easily read, and is essentially a book for the practitioner.

